# New Appliances and Things Medical

**Published:** 1895-02-09

**Authors:** 


### New Appliances and Things Medical.

### ROLLED OATS.—FOOTBALL BRAND.

(Simpson, Roberts, and Co., 40, Stanley Street, Liverpool).

There seems to be a demand for oatmeal in its various forms at the present time, and American and German oatmeals are being largely advertised in this country. Notwithstanding these importations, English oats fetch a higher price than foreign, and we understand that in the Football Brand of rolled oats advantage is taken of the superiority of the English ground cereal. The rolled oats are prepared in a special way, by which the oats are partially cooked and therefore require only a short time to finally cook them into a complete and wholesome food. It is well known that oatmeal, as compared with that derived from other cereals, is far richer in albuminoid compounds and as such deserves to be more generally used. In addition, oats contain in their ash a higher percentage of calcium and other phosphates than wheat or rice, and consequently are to be recommended for children and young persons. The proprietors of Football Oats state that ten minutes boiling is all that is required to cook their preparation, and we have been able to confirm this statement with the sample sent us for examination, though better porridge is produced by longer boiling. Many of the foreign oats contain appreciable quantities of maize and sometimes barley, but from a microscopical examination of the starches present in these rolled oats, we are satisfied that these other cereals are absent, and this fact supports the statement that the oats employed for the preparation of this meal are home grown. The porridge made from the Football Brand oatmeal besides possessing intrinsic merit is quite the pleasantest we have yet met with.

